# Acute Myocarditis Following the Administration of the Second BNT162b2 COVID-19 Vaccine Dose

**DOI:** 10.7759/cureus.18880

**Published:** 2021-10-18

**Authors:** Mohammed A Miqdad, Hamze Nasser, Abdullah Alshehri, Abdul Rahman Mourad

**Affiliations:** 1 Internal Medicine, Dr. Sulaiman Al-Habib Hospital, Khobar, SAU; 2 Cardiology, Dr. Sulaiman Al-Habib Hospital, Khobar, SAU

**Keywords:** sars-cov-2, myopericarditis, myocarditis, covid-19 vaccine, mrna vaccine, bnt162b2

## Abstract

COVID-19 disease has infected millions of people worldwide during the pandemic; hence, the need for an effective and safe vaccine was urgently required. A two-dose of the BNT162b2 mRNA COVID-19 vaccine was reported to have 95% efficacy in preventing COVID-19. The short-term safety profile recorded mild to moderate pain at the injection site, fatigue, and headache. The critical adverse effects were low and similar in the placebo group. However, we report the case of an 18-year-old male who developed acute central crushing chest pain four days following administration of the second dose of the BNT162b2 COVID-19 vaccine. After extensive cardiac workup, including coronary arteries diagnostic angiography, myocarditis was suspected and confirmed by a cardiac MRI. Fortunately, the patient's clinical condition gradually improved in the form of clinical symptoms and laboratory findings. He was discharged after one week of stay in hospital with regular follow-up in the cardiac clinic.

## Introduction

BNT162b2 mRNA is one of the earliest evolved vaccines in response to the coronavirus disease 2019 (COVID-19) pandemic. The administration of BNT162b2 is generally considered safe and associated with mild and self-limiting adverse effects, including pain at the injection site, fever, and chills, especially after the second dose. Moreover, fatigue, headache, muscular and joint pain were noticed, but no serious systematic events were recorded [1].

Nevertheless, since April 2021, there have been increased reports of myocarditis and pericarditis following the administration of mRNA COVID-19 vaccination, according to the Centers for Disease Control and Prevention. Further, these cases are rare, and most patients were fully recovered with conservative management. Concerning symptoms that should be immediately investigated are chest pain, shortness of breath, or palpitation. All suspected myopericarditis cases related to the COVID-19 vaccines must be reported to the Vaccine Adverse Event Reporting System [2].

## Case presentation

This is an 18-year-old male with no significant past medical history, presented to the emergency department complaining of central crushing chest pain four days following the second dose of the BNT162b2 COVID-19 vaccine. The pain was aggravated by respiration with no radiation or other associated symptoms. Upon admission, his vital signs were: Temperature 36.6 °C, pulse rate 74 beats/minute, blood pressure 120/65, O2 saturation 99%, and BMI 30.1. Cardiovascular and pulmonary examinations were unremarkable.

**Figure 1:**
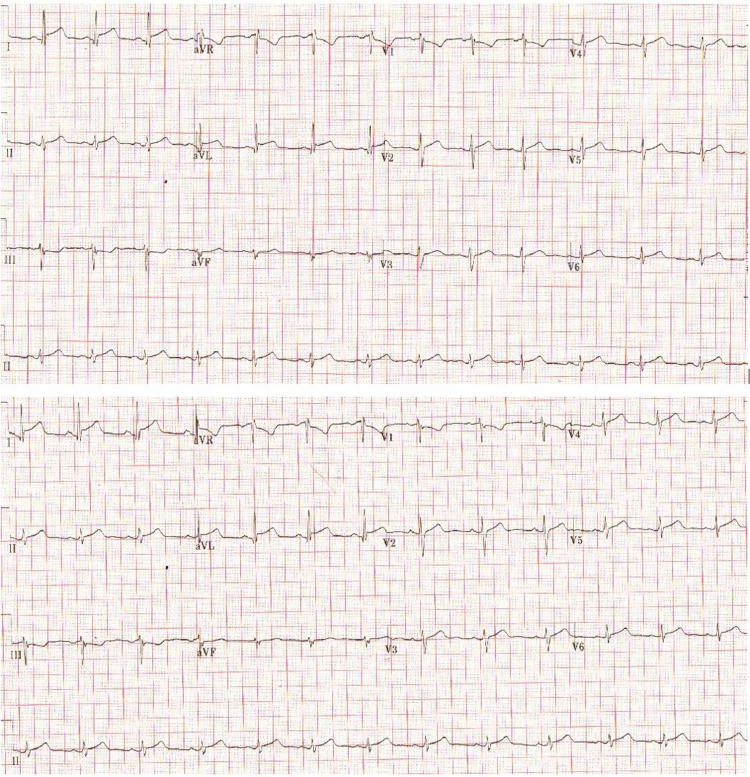
Electrocardiogram Two consecutive ECG showed an ST-elevation in leads I, II along with V2-V6.

**Figure 2:**
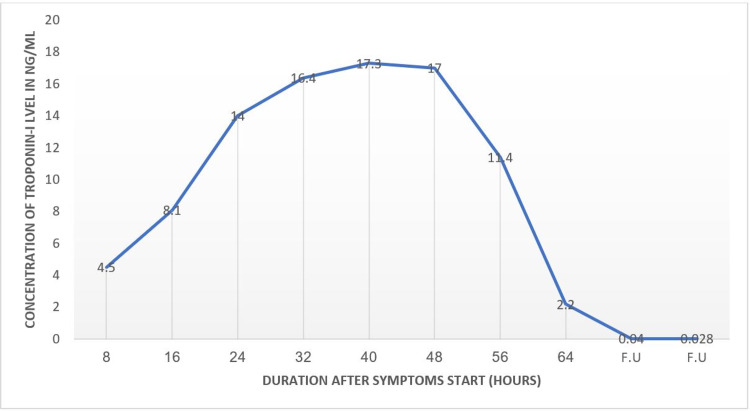
Troponin-I trend during admission F.U.: Follow-up

Initial ECG was normal with no apparent abnormalities, along with routine blood workup (Table 1), aside from mildly elevated high sensitivity troponin-I (2ng/ml) on arrival at the emergency room. However, while the initial ECG was unremarkable, the following ECG started to show ST-segment elevation (Figure 1) together with a gradual troponin-I elevation (Figure 2). Hence, the patient was admitted to the cardiac care unit for close cardiac monitoring and further workup, including diagnostic coronary angiography.

**Table 1:** Laboratory investigations HGB: Hemoglobin; WBC: White blood count; CK-MB: Creatine kinase-MB; BNP: Brain natriuretic peptide; TSH: Thyroid stimulating hormone; CPK: Creatine phosphokinase; CRP: C-reactive protein; T.bilirubin: Total bilirubin; ESR: Erythrocyte sedimentation rate; ALT: Alanine transaminase; AST: Aspartate transaminase; ALP: Alkaline phosphates.

HGB	13.9 g/dL	CK-MB	10.60 ug/L (H)	ESR	18 mm/hour
WBC	7.81 × 10^9^/L	Urea	3.10 mmol/L	ALT	50 IU/L (H)
Platelet	340 × 10^9^/L	Creatinine	63.3 umol/L	AST	101 IU/L (H)
Na	141 mmol/L	BNP	< 10.0 pg/ml	ALP	66 IU/L
K	4.1 mmol/L	D-dimer	0.35 mg/L FEU	CRP	42 mg/L
Mg	0.73 mmol/L	TSH	1.51 mIU/L	T. bilirubin	7 umol/L
Ca	2.33 mmol/L	CPK	280 IU/L (H)	Procalcitonin	0.05 ng/ml

**Figure 3:**
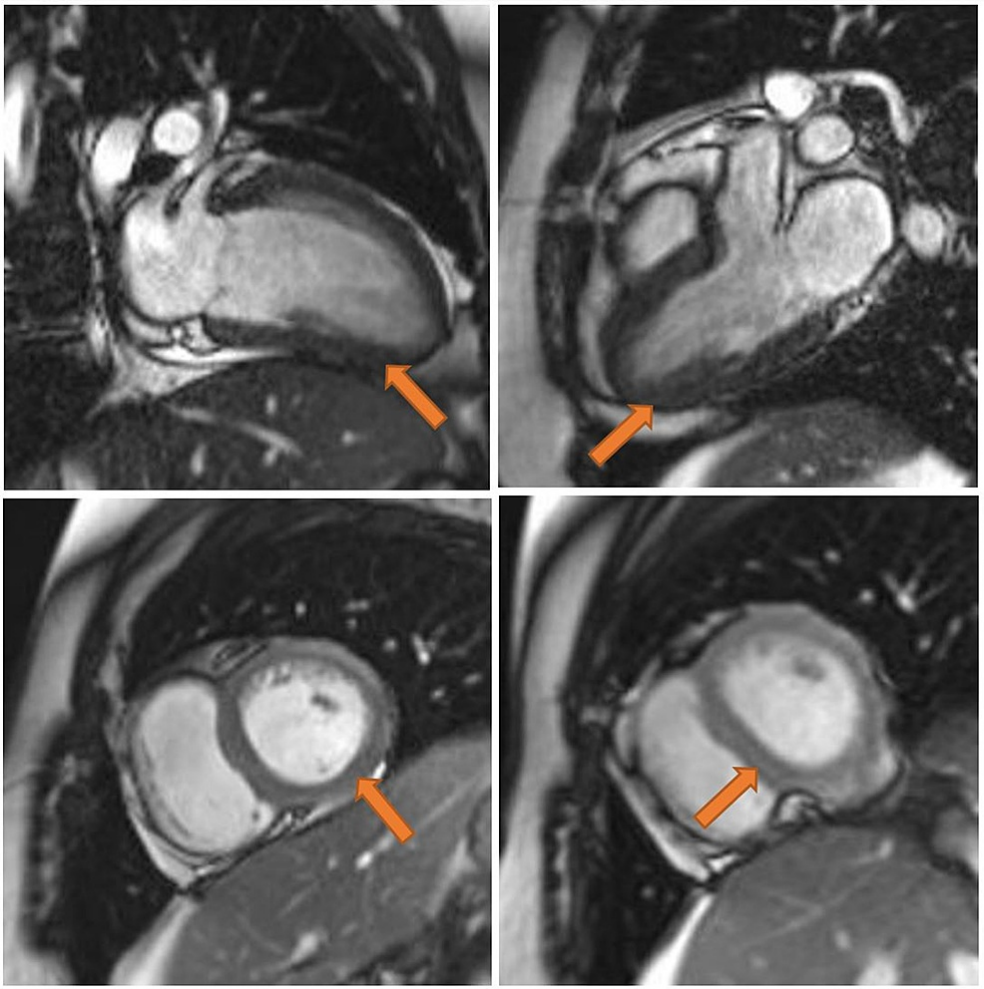
Cardiac MRI There is diffuse left ventricular myocardial edema with associated subpericardial enhancement pattern on delay acquisition. There is also severe global mild hypokinesia with no evidence of pericarditis or pericardial effusion.

Consequently, echocardiography showed normal global systolic left ventricular function with an estimated ejection fraction of 63%, along with normal right ventricular function and no valvular abnormalities. Diagnostic angiography revealed normal coronary arteries. Therefore, he was started on aspirin anti-inflammatory dose, colchicine, and proton pump inhibitors for a preliminary diagnosis of myocarditis. Additionally, cardiac MRI confirmed the diagnosis of myocarditis (Figure 3), with an estimated ejection fraction of 55%. He was accordingly initiated on low dose ramipril and discharged after one week of observation on the anti-inflammatory dose of aspirin with gradual tapering, along with colchicine and protective proton pump inhibitors. While the patient continued to improve in terms of clinical symptoms and laboratory findings, an endomyocardial biopsy was not warranted.

Upon follow-up in the cardiology clinic, his symptoms completely resolved, and troponin-I returned to normal. Follow-up echocardiography revealed a normal left ventricular ejection fraction and ramipril was discontinued accordingly.

## Discussion

Acute myocarditis following the second dose of the BNT162b2 mRNA COVID-19 vaccine was reported in five cases, while one case was reported after the first dose. Similar to our case, all patients were mildly symptomatic and managed with non-steroidal anti-inflammatory drugs (NSAIDs) and colchicine. Further, cardiac MRI was the diagnostic modality that showed features consistent with myocarditis [3]. Likewise, acute myocarditis was reported in a young male six hours after the second dose of the BNT162b2 vaccine, for which he was managed conservatively and entirely recovered [4]. In both case reports, those patients were discharged approximately one week after admission with no lethal consequences [3-4].

Another case series described seven adolescents who developed myocarditis or myopericarditis four days following the second dose of the BNT162b2 mRNA COVID-19 vaccine. Similarly, the suspected diagnosis of vaccine-related myopericarditis was proposed after cardiac MRI and excluding other common etiologies, especially infectious origin. Additionally, six patients were treated with NSAIDs, four had received intravenous immune globulin (IVIg), and one received high-dose methylprednisolone [5].

Ordinarily, myocarditis following immunization is considered an unusual adverse effect; however, it was reported after the smallpox vaccine in adults with a documented incidence of 1:10,000 [3]. Moreover, cardiovascular complications related to COVID-19 disease were reported in 20-30% of hospitalized patients and was associated with poor outcome [3, 6]. COVID-19-induced myopericarditis has been documented in multiple case reports and literature reviews. For instance, a previously healthy woman had developed acute myopericarditis with cardiac tamponade secondary to COVID-19 disease [7]. The pathophysiological mechanism of COVID-19-induced myopericarditis is thought to be related to direct cardiomyocytes damaged by the virus, the host immune response of cytotoxic T lymphocytes, and the effect of inflammatory cytokines storm [3]. Therefore, the latter theories may explain the association between the mRNA COVID-19 vaccine and myopericarditis.

Our patient received an anti-inflammatory dose of aspirin (600mg every 6 hours) and tapered down gradually. Besides, low-dose ramipril was initiated based on the borderline ejection fraction; nevertheless, ramipril was omitted upon follow-up after restoring the normal cardiac contractility and ejection fraction. Finally, we suggested that acute myocarditis might be an adverse outcome to the BNT162b2 mRNA vaccine in our case. We recommend that our findings be interpreted cautiously as no strong evidence clarifies the association between myopericarditis and the BNT162b2 mRNA vaccine.

## Conclusions

In response to the Centers for Disease Control and Prevention, all suspected myopericarditis related to COVID-19 vaccines must be reported to the Vaccine Adverse Event Reporting System. Although myopericarditis can be fulminant and lethal, most reported cases related to the mRNA vaccine were managed conservatively with no serious complications. Clinical manifestations concerning myopericarditis, including chest pain or heaviness, palpitation, or shortness of breath, warrant immediate attention to rule out the possibility of myopericarditis, especially in the era of COVID-19 vaccination.
